# 

**Published:** 1872-09

**Authors:** 


### CONTENTS:

ORIGINAL COMMUNICATIONS:
- ARTICLE I. St. Luke's Hospital. Ovariotomy — Perforation of Intestines — Death ..... 513
- ARTICLE II. Gunshot Wound of Stomach and Kidney — Recovery. By J. W. Brooks, M.D., Chicago .. 519
- ARTICLE III. Lateral Dislocation of the Knee Joint — Rapid Recovery. By L. P. Griffin, M.D., Chicago ... 520
- ARTICLE IV. Lecture on Chloral Hydrate. By J. H. Etheridge, M.D. Chicago ..... 521
- ARTICLE V. The Panaritium (Felon) — Consequences and Treatment. By Carl Proegler, M.D., Addison, Ill. .. 527

SELECTIONS:
- On the Application of Gases as a Means of Destroying Contagion ... 530
- Cerebro-Spinal Meningitis ..... 538
- Membranous Formations on Mucous Surfaces ..... 545
- The Science of Meteorology, etc. ..... 550
- Hydrophobia ..... 557
- Hysteria ..... 562

EDITORS' BOOK TABLE ..... 571

EDITORIAL ..... 573

